# Complete Genome Sequence of the *Pantoea* Phage AH07

**DOI:** 10.1128/MRA.00819-21

**Published:** 2021-11-04

**Authors:** Sam J. Roth, Greg P. Krukonis, Véronique A. Delesalle

**Affiliations:** a Department of Biology, Gettysburg College, Gettysburg, Pennsylvania, USA; b Department of Biology, Angelo State University, San Angelo, Texas, USA; Portland State University

## Abstract

Bacteriophages of the phyllosphere have not been extensively described, despite their role in bacterial communities on this plant organ. Here, we describe a temperate *Pantoea* phage, AH07, that was isolated from the leaves of horse chestnut trees. The 37,859-bp linear double-stranded DNA genome contains 58 putative genes, including an integration cassette.

## ANNOUNCEMENT

As part of a broad evolutionary research program (1–5), bacteriophages from the leaves of horse chestnut trees (*Aesculus hippocastanum*; Sapindaceae) from Angel and Greyhound Meadow (Oxford, UK) were isolated in 2011 to 2014 on bacterial strains that themselves were isolated from these leaves (1). Bacterial isolates were assigned to a given genus and, if possible, species based on sequencing of approximately 800 bp of the 16S rRNA region and the top BLASTn hit associated with a sequence (E value of <10^−10^) (1). Individual phages were each single plaque purified at least three times on their focal hosts and amplified by overnight culturing in 10 ml King’s broth with 100 μl of isolation bacteria (1); we refer to these as phyllophages AH01 to AH07. Here, we describe the temperate phage AH07, which was isolated on a *Pantoea* species.

The culture lysate was filtered (0.45 μm), and DNA extraction was performed by the Koskella laboratory following the protocol for the Wizard PCR Preps DNA purification system kit (product no. 7170; Promega). Library preparation and sequencing were performed at the North Carolina State University Genomic Science Laboratory. Libraries were prepared using an Illumina TruSeq Nano DNA library preparation kit, following the manufacturer’s protocol. Sequencing was done with an Illumina MiSeq platform, using a v3 single-end 150-bp flow cell. Genome assembly and annotation were performed at Gettysburg College. The 409,887 single-end 150-bp reads were aligned and assembled into one contig with 1,624× coverage using GS *De Novo* Assembler v2.9; all untrimmed reads were used, and contig quality was verified in Consed v29 (6, 7). Genome ends were determined with PAUSE (https://cpt.tamu.edu/analysis-with-pause3-2016-edition) and PhageTerm (8). The finished sequence was imported into DNA Master v5.22.22 (http://cobamide2.bio.pitt.edu/computer.htm) to map open reading frames. Putative genes were called based on both Glimmer v3.0 and GeneMark v2.5 algorithms (9, 10). Putative functions of gene products were predicted using BLAST v2.12 (11) and HHpred (12). For BLASTp matches, an E value of <10^−5^ was required to assign function. For HHpred matches, a high probability (>85%), substantial coverage (>50%), and low E value (<10^−5^) were required. Default settings were used with all of the aforementioned programs.

AH07 has a 37,859-bp genome with a GC content of 52.2%, 58 putative genes, including 39 genes with assigned functions, and no tRNA genes. The genome architecture of AH07 follows the common organization of *Siphoviridae* phages, with an ordered cluster of structural genes followed by a mixture of genes involved in DNA metabolism or of unknown function (13). The integration cassette (tyrosine integrase, excise, and immunity repressor) follows the structural genes, and the lysis cassette (holin, endolysin, and Rz-like lysis protein) is located at the end of the genome. A phage isolate obtained from a different leaf on the same tree in 2012 was sequenced following the protocols described above and was determined to be identical to AH07. From whole-genome searches by BLASTn (Table 1), AH07 was found to have the greatest nucleotide similarity to segments of Pantoea agglomerans and Pantoea vagans genomes, supporting its temperate life cycle. When the BLASTn search was restricted to phages (Table 1), the best matches were to *Siphoviridae* samples, intriguingly isolated from throughout the human body (14).

**TABLE 1:** Results of whole-genome (nucleotide) searches for AH07 using BLASTn

Database	Sample description^*a*^	Query coverage (%)	Identity (%)	Accession no.
Complete	Pantoea agglomerans strain CFSAN047153	69	89.64	CP034469.1
Complete	Pantoea agglomerans strain CFSAN047154	69	89.64	CP034474.1
Complete	Pantoea vagans strain PV989	62	92.44	CP028349.1
Complete	Pantoea agglomerans strain ASB05	49	89.64	CP046722.1
Restricted to phages	TPA asm: *Siphoviridae* sp. strain ct4sp3	58	93.94	BK015502.1
Restricted to phages	TPA asm: *Siphoviridae* sp. isolate ctTyS5	48	87.33	BK029822.1

a The complete genome of AH07 was compared to the complete nucleotide database or the same database restricted to tailed phages (taxid 10699, 10662, and 10744). For each search, the best matches are reported (defined as more than 45% query coverage). The percentage of the query covered by alignment to the database sequence and the percent identity of matched alignment are reported. TPA asm, third-party annotation assembly.

### Data availability.

The genome sequence and associated information can be found under GenBank accession no. MZ501270, SRA accession no. SRX11736858, and BioProject accession no. PRJNA754193.
